# Assessment of Repetitive and Compulsive Behaviors Induced by Pramipexole in Rats: Effect of Alpha-Synuclein-Induced Nigrostriatal Degeneration

**DOI:** 10.3390/biomedicines10030542

**Published:** 2022-02-24

**Authors:** Mélina Decourt, Eric Balado, Haritz Jiménez-Urbieta, Maureen Francheteau, Pierre-Olivier Fernagut, Marianne Benoit-Marand

**Affiliations:** Laboratoire de Neurosciences Expérimentales et Cliniques, INSERM, Université de Poitiers, 86000 Poitiers, France; melina.decourt@univ-poitiers.fr (M.D.); eric.balado@univ-poitiers.fr (E.B.); haritzjimenez@hotmail.com (H.J.-U.); maureen.francheteau@univ-poitiers.fr (M.F.); marianne.benoit.marand@univ-poitiers.fr (M.B.-M.)

**Keywords:** Parkinson’s disease, impulse control disorders, pramipexole, substantia nigra, hoarding, post-training signal attenuation

## Abstract

Treatment with dopamine agonists in Parkinson’s disease (PD) is associated with debilitating neuropsychiatric side-effects characterized by impulsive and compulsive behaviors. The vulnerability to develop such impairments is thought to involve interactions between individual vulnerability traits, types of antiparkinsonian medications, and the neurodegenerative process. We investigated the effect of the dopamine D3/D2 agonist pramipexole (PPX) and selective nigrostriatal degeneration achieved by viral-mediated expression of alpha-synuclein on the expression of repetitive and compulsive-like behaviors in rats. In a task assessing spontaneous food hoarding behavior, PPX increased the time spent interacting with food pellets at the expense of hoarding. This disruption of hoarding behavior was identical in sham and lesioned rats. In an operant post-training signal attenuation task, the combination of nigrostriatal lesion and PPX decreased the number of completed trials and increased the number of uncompleted trials. The lesion led to an increased compulsive behavior after signal attenuation, and PPX shifted the overall behavioral output towards an increased proportion of compulsive lever-presses. Given the magnitude of the behavioral effects and the lack of strong interaction between PPX and nigral degeneration, these results suggest that extra-nigral pathology may be critical to increase the vulnerability to develop compulsive behaviors following treatment with D3/D2 agonists.

## 1. Introduction

Chronic treatment with dopamine agonists can lead to a number of cognitive and neuropsychiatric side-effects in a significant proportion of patients with Parkinson’s disease (PD). These non-motor side-effects that essentially occur in patients treated with D3/D2 dopamine agonists include different forms of impulsive-compulsive behaviors described as the failure to resist the urge to behave repetitively and compulsively in ways that can be hazardous for oneself or other people and interfere with daily living activities [1,2]. These behaviors are defined as behavioral addictions such as compulsive gambling, shopping, eating, or sexual behavior. Other impulsive compulsive behaviors include “punding” (repetitive, stereotyped, and purposeless behavior), hobbyism (repeated and long-lasting execution of preferred activities such as leisure or work) [3], as well as hoarding behavior [4]. Since these behaviors are also reported in patients treated with D3/D2 agonists for other non-neurodegenerative conditions such as restless legs syndrome [5] or prolactinoma [6], the respective contributions of the drug and of dopaminergic neurodegeneration remain poorly understood. Rodent models of PD recapitulate a wide array of cognitive and neuropsychiatric impairments that occur in PD (for review, see [7]) and are also amenable to investigate the pathophysiology of non-motor side-effects of dopamine replacement therapy such as impulsive-compulsive behaviors. Several studies have thus demonstrated that nigrostriatal degeneration decreases various dimensions of inhibitory control [8,9,10] and that PPX could aggravate these deleterious effects of the lesion [8,9]. Conversely, in tasks assessing risky decision making, the ability of D3/D2 agonists to increase risk-taking behavior was not altered by a nigrostriatal lesion [11,12]. In PD patients, behaviors such as punding, hobbyism, or hoarding involve repetitive and stereotypic sequences of simple or complex actions performed without goal and reward. In other impulsive-compulsive behaviors such as pathological gambling, impaired learning from reward, and loss outcomes lead to persistence of the maladaptive behavior [2]. Perseverance of the behavior in spite of lack of reward is thus a common process in all impulsive-compulsive behaviors experienced by the patients.

In rodents, both spontaneous behaviors and operant conditioning procedures can be used to investigate how nigrostriatal degeneration and treatment with D3/D2 agonists may affect behaviors that are relevant to those occurring in PD patients. As the behavioral repertoire of rodent involves many repetitive behaviors (food hoarding, nest building, grooming), the assessment of these behaviors can easily be implemented in laboratories. Conversely, operant conditioning procedures require dedicated equipment and training, but allow for the assessment of more complex behaviors and cognitive processes [13]. For instance, the post-training signal attenuation (PTSA) task can be used to investigate the processing of stimulus-outcome associations and their devaluation [14].

In the present study, we tested the hypothesis that the respective contributions of nigrostriatal degeneration, treatment with dopamine agonists, as well as lesion and drug interactions in the emergence of impulsive-compulsive behaviors could be identified using behavioral procedures that can lead to perseverative and compulsive behaviors. To this end, we investigated the effect of the D3/D2 agonist pramipexole (PPX) on repetitive and perseverative behaviors using either spontaneous behavior in a food hoarding task, or operant responding in the PTSA task in normal rats and in animals with alpha-synuclein-induced degeneration of nigrostriatal dopaminergic neurons.

## 2. Materials and Methods

### 2.1. Animals

All experiments were approved by the Comethea Poitou-Charentes (C2EA-84, project approval #24114-2020021310245838) and performed under the European Directive (2010/63/EU) on the protection of animals used for scientific purposes. Male Sprague Dawley rats (*n* = 61, 175–200 g upon arrival, Janvier, France) were housed by pair on a reversed 12 h light/dark cycle (lights on at 20h00). After seven days of habituation, they were isolated and food restricted to 15 g per day (two days before the hoarding task and one week before the PTSA task). Water was available *ad libitum*. Experiments were performed on 7 sham and 8 lesioned rats for the hoarding task (experiment 1) and on 22 sham (12 saline, 10 PPX) and 24 lesioned (13 saline, 11 PPX) rats for the PTSA test (experiment 2).

### 2.2. Hoarding Task (Experiment 1)

**Figure 1:**
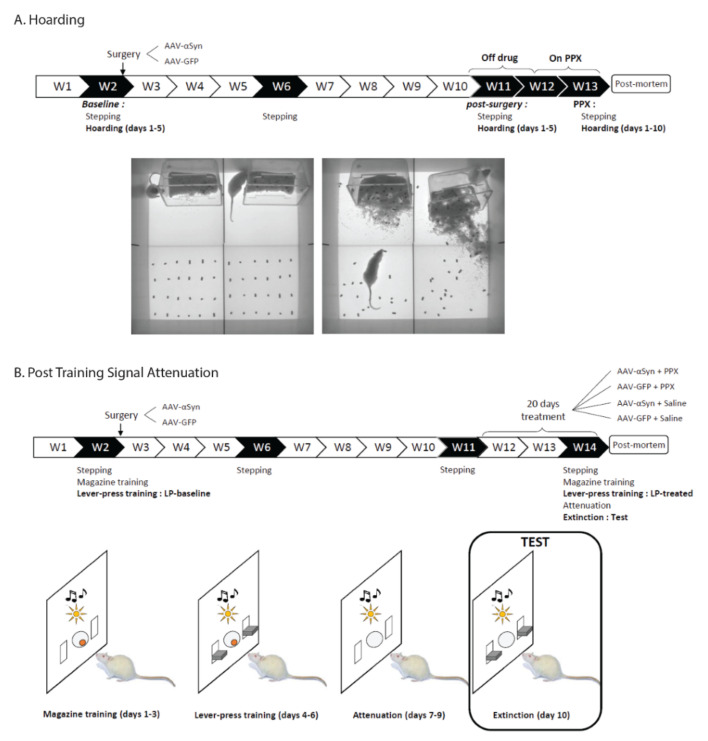
Experimental design. (**A**) Timeline of experiment 1 (hoarding). After baseline assessment of motor function (stepping) and of hoarding behavior, bilateral lesion of the substantia nigra *pars compacta* was achieved via viral-mediated bilateral overexpression of α-synuclein. Stepping was performed again at 4 and 9 weeks after surgery. Hoarding behavior was assessed at 9 weeks post-surgery (Off PPX) and at 11 weeks, during 10 days of PPX administration (0.3 mg/kg/day, s.c.). Hoarding behavior was assessed during 30 min session in a 100 × 50 cm open field. Pictures show the configuration of the task at the beginning (left) and at the end of a hoarding session (right). (**B**) Timeline of experiment 2 (Post training signal attenuation task). After baseline assessment of motor function (stepping), rats were trained to collect 45 mg sucrose pellets from the magazine (magazine training), then to press a reinforced lever in order to obtain a 45 mg sucrose pellet reward (Lever-press (LP) training: LP baseline). Bilateral lesion of the substantia nigra *pars compacta* was achieved via viral-mediated bilateral overexpression of α-synuclein and the stepping test was performed again at 4 and 9 weeks after surgery. Animals then received PPX (0.3 mg/kg/day, s.c.) or saline for 20 days. 10 days after the initiation of PPX treatment, magazine training was performed, followed by lever-press training, attenuation, and extinction. During magazine training (days 1–3), both levers were retracted and the occurrence of a compound stimulus (light + tone) indicated reward availability in the magazine. During lever-press training (days 4–6), both levers were available and pressing the reinforced lever triggered the presentation of the compound stimulus and the delivery of a food pellet in the magazine. During attenuation (days 7–9), both levers were retracted, and presentation of the compound stimulus was associated with no reward being delivered in the magazine. During extinction (test, day 10), both levers were available and pressing the reinforced lever triggered the presentation of the compound stimulus associated with no reward delivered in the magazine.

Each hoarding session was performed for 30 min, during 5 consecutive days for baseline, 5 consecutive days for post-surgery time-point (9 weeks after surgery, after progressive development of the lesion), and 10 consecutive days during treatment (Figure 1A). Animals were video-recorded and their movements tracked with VideoTrack system (ViewPoint, Lyon, France). The setup was constituted of an open-field corridor (1 m long × 0.5 m wide × 0.4 m high). The area was divided in two zones of same surface (0.5 m long × 0.5 m wide): the food zone and the home zone. In the food zone, 24 food pellets (of approximately 2 g each) were placed in 4 lines of 6. At the start of each test session, the rat and its home-cage were placed in the home zone with the home-cage placed on its side in a corner of the zone to allow animal access (Figure 1A). The following parameters were analyzed with Solomon Coder software (András Péter, Keele, UK): (1) the number of food pellets collected, (2) the time spent in interaction with the food pellets, (3) the time spent in food pellets zone, and (4) the interaction with food pellets time and time in food pellets zone ratio.

### 2.3. Post Training Signal Attenuation Test (PTSA, Experiment 2)

This test was performed as described before [15] in operant chambers (Med Associates, Fairfax, VT, USA) equipped with two identical retractable levers surmounted by a light cue, a sucrose pellet dispenser, a house light, and a tone. Four stages composed this task (Figure 1B): the magazine training stage (days 1–3), the lever press training stage (days 4–6), the attenuation stage (days 7–9), and the extinction stage (test, day 10).

#### 2.3.1. Magazine Training Sessions

The two levers were retracted, and rats were trained to collect a 45 mg sucrose pellet reward (1118251, TestDiet, Saint Louis, MO, USA) in the pellet dispenser. House light was turned on and a pellet was dropped in the food magazine after 5 s-delay. At the same time, house light was turned off while compound stimulus was turned on (tone and pellet dispenser light) until the rat collected the pellet. Between each trial, an interval of 30 s was applied. This first stage’s day ends after 30 completed trials or after a total of 40 trials.

#### 2.3.2. Lever Press Training Sessions

Rats were trained to associate lever press, composite stimulus, and sucrose reward. To this end, levers are available for pressing, but only one is reinforced (RL). Levers were inserted after 5 s-delay. A press on the RL results in the delivery of one pellet in the pellet dispenser, simultaneous with the composite stimulus. The levers were retracted and the house light was turned off after 15 s or after pellet collection. A press on the non-reinforced lever (NRL) had no consequences. Intertrial intervals of 30 s were applied. On day 4, rats were trained until 24 completed trials or until performing 60 trials. If rats performed less than 20 trials within the session, they were allowed to perform another session at the end of day. On day 5 and 6, rats were trained until 40 trials were successfully completed or until animals reached 60 trials.

#### 2.3.3. Attenuation Sessions

For 30 trials, the rats were exposed to the same configuration as during magazine training sessions except that no food reward was delivered.

#### 2.3.4. Extinction Session (Test)

The levers were available as during the lever press training sessions, but no food reward was delivered in any case. Lever press on RL induced only the compound stimulus. The session lasted for 50 trials.

At baseline, only magazine training and lever press training sessions were performed, the full exercise was realized only during PPX/saline treatment. The following parameters were recorded: (1) the number of presses after the first press on the RL (extra lever press, ELP) in completed trials (ELP-C) and in uncompleted trials (ELP-U), (2) number of lever presses on RL, (3) the number of lever presses on NRL.

### 2.4. Alpha-Synuclein-Mediated Lesion and Assement of Akinesia

Rats were anesthetized with isoflurane and placed in a stereotaxic frame (Kopf Instruments^®^, Tujunga, CA, USA). Pre-surgical analgesia was performed with ketoprofen (10 mg/kg, i.p.) and local analgesia with 2% xylocaine gel. Two bilateral injections were performed in the substantia nigra *pars compacta* (SNc) (in millimeters from bregma and dura, according to Paxinos and Watson rat brain atlas: AP: −5.1 and −5.6; ML: ±2.2, and ±2; DV: −8) with 1 µL of AAV2 expressing human A53T mutant α-synuclein (CMVie/SynP-synA53T-WPRE, 5.2 × 10^13^ gcp/mL) at 0.2 µL/min [8,16,17]. Control group was injected with 1 µL of AAV2 expressing GFP (green fluorescent protein) (CMVie/SynP-GFPdegron-WPRE, 3.7 × 10^13^ gcp/mL). Post-surgical analgesia was performed with ketoprofen (10 mg/kg/day, i.p.) for two days. Akinesia was estimated twice a day during four consecutive days with the stepping test at baseline, 4 and 9 weeks after surgery. To this end, the hindlimbs were held, the hind part was raised above the surface of the bench to and the forelimb not tested was fixed by the experimenter. The number of adjusting steps was counted for each paw in the backhand and forehand direction of the movement, as previously described [18] and averaged for analysis.

### 2.5. Treatment

PPX (Sequoia Research Products, Berkshire, UK) was prepared daily in sterile saline solution and administrated subcutaneously at a dose of 0.3 mg/kg/day for 10 days (experiment 1) or 21 days (experiment 2). This dose was chosen based on its previously demonstrated ability to improve motor deficits in rat models of PD [8,11], and for its lack of side-effects such as stereotypies [19]. Behavioral tests were performed 30 min after injection of PPX or vehicle (sterile saline).

### 2.6. Tissue Processing and Histopathological Analysis

A sublethal dose of sodium pentobarbital (120 mg/kg, i.p.) was injected prior to performing an intracardiac perfusion with 200 mL 0.9% NaCl followed by 200 mL ice-cold 4% formaldehyde. Brains were post-fixed overnight in 4% formaldehyde and cryopreserved in H_2_O/20% sucrose solution at 4 °C for two days. They were then frozen in isopentane at −40 °C and stored at −80 °C until further processing. Serial 50 µm coronal free-floating sections were collected in a cryoprotectant solution and stored at −20 °C.

#### 2.6.1. Immunohistochemistry

After 3 washes in Tris Buffer Saline 1X (TBS, 15 min each), sections were incubated 10 min in H_2_O_2_ solution (S202386-2, Agilent Technologies, Santa Clara, CA, USA) to quench endogenous peroxidases. After 3 washes, an incubation of 90 min in a blocking solution containing 3% BSA (bovin serum albumin) and 0.3% Triton in TBS 1X was performed. Sections were then incubated 24 h at 4 °C with mouse anti-TH (1/5000, MAB318, Sigma-Aldrich, Saint Louis, MO, USA) in blocking solution. Sections were washed again 3 times in TBS 1X, and then incubated 1 h at room temperature (RT) with EnVision HRP system anti-mouse (K400111-2, Agilent Technologies, Santa Clara, CA, USA) in blocking solution. Then, sections were washed again three times in TBS 1X and immunoreactions were revealed with DAB peroxidase substrate (K346811-2, Agilent Technologies, Santa Clara, CA, USA). Finally, sections were mounted on gelatin-coated slides and coverslipped with DePeX.

#### 2.6.2. Stereology

TH-positive neurons were counted using the optical fractionator method on every 6th section of the SNc as previously described [8]. Systematic random sampling was performed with the Mercator Pro V6.5 (Explora Nova, La Rochelle, France) software coupled with a Leica 5500B microscope. Following delineation of the SNc with ×5 objective, counting was done with ×40 objective.

### 2.7. Statistical Analysis

Data are expressed as mean ± Standard Error of the Mean and analyzed using GraphPad Prism-9 software. Normality was tested by the Kolmogorov–Smirnov test. Data having a Gaussian distribution were analyzed using one-way or two-way analysis of variance (ANOVAs) followed by Tukey’s post-hoc test for multiple comparisons. Greenhouse-Geisser correction for sphericity was applied whenever appropriate. Student’s *t*-test was used for comparisons between two groups. Mann–Whitney test or Kruskal–Wallis test followed by Dunn’s post-hoc test were applied whenever appropriate when samples did not follow a normal distribution or variance equality. For all analyses, a *p* value < 0.05 was considered significant.

## 3. Results

### 3.1. α-Synuclein-Induced Degeneration of Nigrostriatal TH Positive Neurons and Motor Impairment

**Figure 2:**
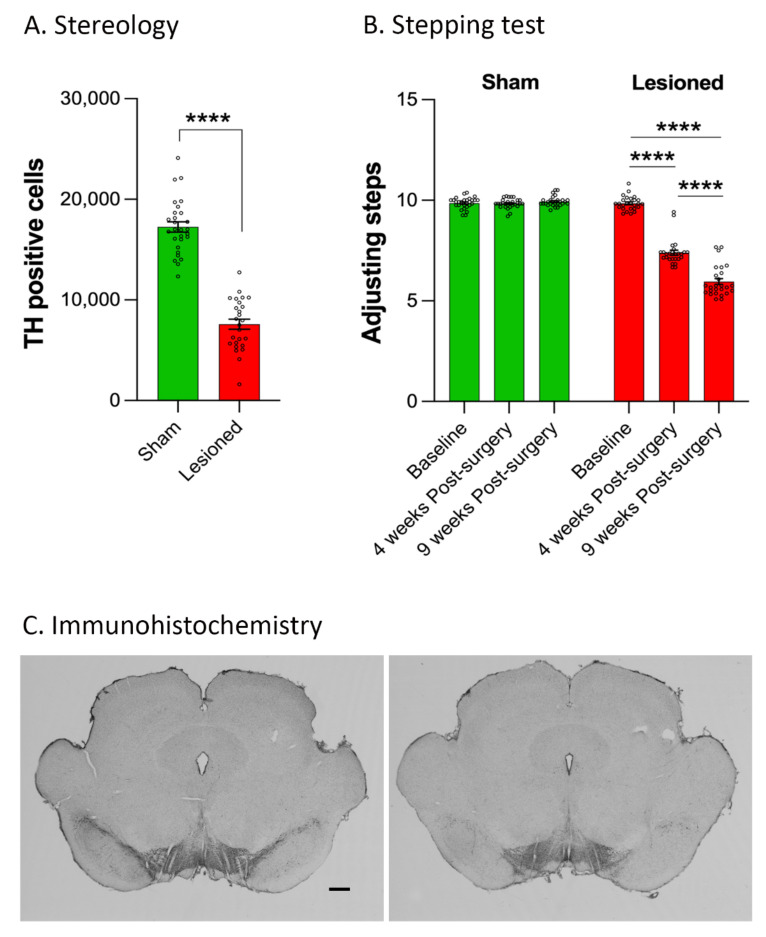
Alpha-synuclein-induced neurodegeneration and motor deficits. (**A**). Stereological counts of the numbers of tyrosine hydroxylase (TH) positive neurons in the substantia nigra *pars compacta* in animals injected with an AAV expressing the green fluorescent protein (GFP, Sham) or human A53T α-synuclein (Lesioned). ****, *p* < 0.0001. (**B**). Motor performances as assessed with the stepping test at baseline, 4 and 9 weeks after surgery. (**C**). Representative images of TH immunohistochemistry in Sham (left panel) and lesioned animals (right panel). Scale bar = 500 µm.

Dopaminergic cell loss induced by α-synuclein expression was assessed by stereological counts of TH positive neurons in the SNc. Viral-mediated expression of α-synuclein led to a significant loss of TH positive neurons (*p* < 0.0001, Figure 2A,C) accompanied by progressive bilateral motor deficits (lesion: F_(1,52)_ = 724.1, *p* < 0.0001; Time: F_(1.770,92.05)_ = 268.3, *p* < 0.0001; Lesion X Time interaction: F_(2,104)_ = 285.8, *p* < 0.0001, Figure 2B). These deficits were significant at 4 weeks (*p* < 0.0001) and worsened 9 weeks following surgery (*p* < 0.0001). As α-synuclein lesioned rats from both experiments displayed a similar degree of lesion (F_(1,51)_ = 1.642, *p* = 0.22) and motor impairment (F_(1,51)_ = 0.76, *p* = 0.39), results are shown for the whole population.

### 3.2. Hoarding (Experiment 1)

**Figure 3:**
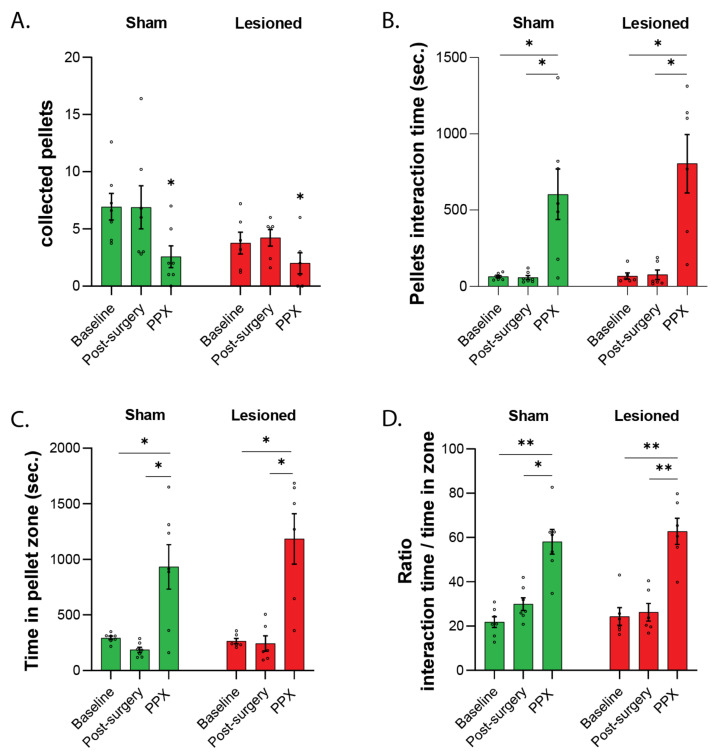
Hoarding (experiment 1). (**A**). Number of food pellets collected in the home cage at baseline, 9 weeks post-surgery (Off drug) and after 10 days of treatment with PPX. *, *p* < 0.05 vs. post-surgery. (**B**). Time spent performing interactions with food pellets. *, *p* < 0.05; **, *p* < 0.01. (**C**). Time spend in the zone containing food pellets. *, *p* < 0.05. (**D**). Ratio of the time spent interacting with food pellets over the time spent in the food pellet zone. *, *p* < 0.05; **, *p* < 0.01.

Hoarding is a spontaneous behavior exhibited by most rodents including rats and is sensitive to alterations of dopamine homeostasis [20,21]. After post-mortem analysis, two lesioned rats were excluded due to a lack of nigrostriatal lesion. Accordingly, data are presented for seven sham and six lesioned rats. Viral-mediated expression of α-synuclein had no significant effect on the different parameters analyzed during the hoarding task, while PPX induced several changes in hoarding behavior (Figure 3). The number of pellets collected was markedly decreased after PPX treatment (F_(1.95,23.41)_ = 8.19, *p* < 0.01), both in sham (−63%, *p* < 0.05) and lesioned rats (−53%, *p* < 0.05) compared to post-surgery values (Figure 3A). In both sham and lesioned groups, PPX induced a marked increase in the time spent interacting with food pellets (F_(1.011,11.13)_ = 25.98, *p* < 0.001, in both sham and lesioned rats, *p* < 0.05, Figure 3B), in the time spent in the food pellets zone (F_(1.041,11.46)_ = 28.68, *p* < 0.001, in both sham and lesioned rats, *p* < 0.05, Figure 3C), and in the ratio of interaction time over time in food pellets zone (F_(1.247,13.71)_ = 62.60, *p* < 0.0001, both in sham (*p* < 0.05), and lesioned rats (*p* < 0.01), Figure 3D). These results indicate that PPX significantly disrupted hoarding behavior, regardless of nigrostriatal denervation.

### 3.3. PTSA (Experiment 2)

Joel et al. [15,22] tested the production of compulsive-like behavior in rats by developing a model based on the deficit of response feedback associated with outcomes of goal-directed responses. Such default in response feedback is achieved by extinguishing the contingency between a compound stimulus and reward availability, leading to a pattern of lever-press responding analogous to compulsive behavior. In the test phase, when lever-presses are not rewarded, rats increase excessive lever-press followed by magazine entry, as in a regular extinction procedure, (extra lever-presses in completed trials; ELP-C). However, the signal attenuation phase in the PTSA paradigm elicits more lever-presses that are not followed by magazine visit (extra lever-presses in uncompleted trials; ELP-U) that represents a form of compulsive behavior [14,23,24]. We measured the number of trials on which the rat attempted to collect the food reward (completed trials) and the number of trials in which rats did not attempt to collect the reward (uncompleted trials), as well as ELP-C and ELP-U to assess the effect of SNc dopaminergic neurons lesion and PPX treatment on compulsive behavior [14,22,25,26].

Experiments were performed on 22 sham (12 saline, 10 PPX) and 24 lesioned (13 saline, 11 PPX) rats. One sham rat was excluded for failing to achieve the cut-off of 20 completed trials during lever-press training [22]. Four lesioned rats were excluded due to a lack of nigrostriatal lesion (*n* = 2) or failing to achieve the cut-off of 20 completed trials during lever-press training (*n* = 2). Accordingly, data are presented for 21 sham (*n* = 12 saline, *n* = 9 PPX) and 20 lesioned rats (*n* = 13 saline, *n* = 7 PPX).

**Figure 4:**
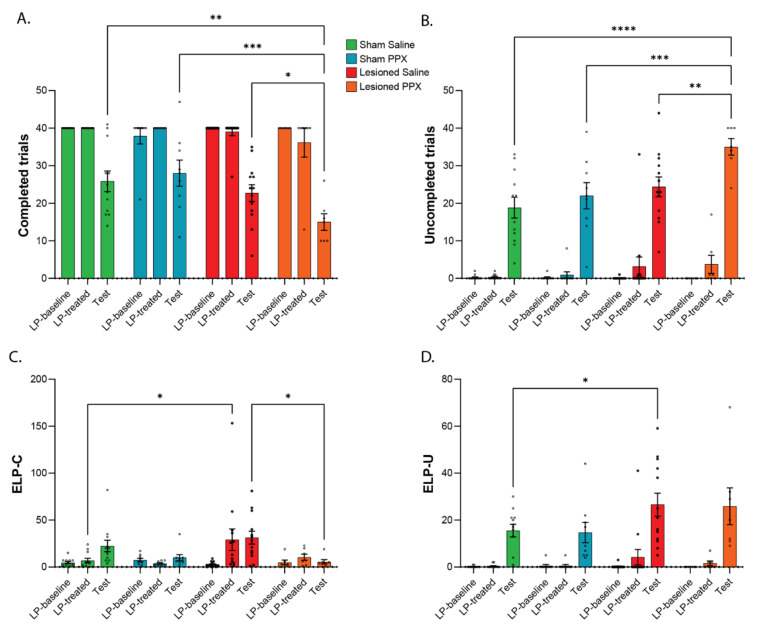
Post training signal attenuation task (experiment 2). (**A**). Number of completed trials during the different stages of the experiment (LP: Lever-press). (**B**). Number of uncompleted trials during the different stages of the experiment. *, *p* < 0.05; **, *p* < 0.01; ***, *p* < 0.001; **** *p* < 0.0001. (**C**). Number of extra lever-press followed by magazine entry (extra lever-presses in completed trials; ELP-C). (**D**). Number of extra lever-press not followed by magazine entry (extra lever-presses in uncompleted trials; ELP-U). *, *p* < 0.05.

All remaining rats performed consistently in completing trials after surgery compared to baseline (F_(1,37)_ = 0.61, *p* = 0.44). On the test stage, the extinction procedure resulted in a decrease of the number of completed trials in all groups (F_(2,74)_ = 103.9, *p* < 0.0001, Figure 4A). However, the magnitude of this decrease was different between groups (F_(6,74)_ = 2.5, *p* < 0.05) and was stronger in lesioned rats treated with PPX compared to all other groups (Figure 4A). During the test stage, the number of uncompleted trials was markedly increased in all groups (F_(2,74)_ = 228.3, *p* < 0.0001, Figure 4B). As for completed trials, the combination of nigrostriatal lesion and PPX treatment also increased the number of uncompleted trials (F_(6,74)_ = 3.59, *p* < 0.01).

To further discriminate between the rats’ response to the encounter of no reward and the rats’ response to the encounter of an attenuated signal, we measured respectively ELP-C and ELP-U as previously described [14]. The number of ELP-C across the stages of the task was different between groups (F_(6,74)_ = 3.55, *p* < 0.01). SNc dopaminergic lesion induced an increase of ELP-C during the lever press training following surgery and treatment (*p* < 0.001 LP-treated vs. LP-baseline, Figure 4C). Moreover, whereas saline-treated sham rats showed an increase in ELP-C on the test stage (*p* < 0.05 Test vs. LP-baseline), dopaminergic lesion of the SNc did not further alter this parameter on test stage compared to LP-treated stage. Furthermore, the increase in ELP-C observed in saline-treated sham and lesioned groups during the test stage was not found in animals treated with PPX (Figure 4C). The ELP-U responses were increased in all groups on test stage (F_(2,74)_ = 58.75, *p* < 0.0001, Figure 4D), indicating that all groups displayed some compulsive-like lever-pressing behavior. However, this effect was stronger in lesioned rats than in sham rats (*p* < 0.05), but PPX treatment did not further affect the number of ELP-U in any of the groups (Figure 4D). To compare overall behavioral outputs during the test phase, the numbers of ELP-C and ELP-U within each group were also analyzed. There was a similar number of ELP-C and ELP-U in sham animals treated either with saline or PPX and in lesioned rats treated with saline. However, the number of ELP-U was significantly higher than the number of ELP-C in lesioned rats treated with PPX (*p* < 0.01), indicating that the combination of nigrostriatal denervation and PPX treatment promoted the expression of compulsive lever-press.

## 4. Discussion

Impulsive-compulsive behaviors are increasingly recognized as a major issue related to the chronic treatment with dopamine agonists in PD and other conditions such as restless leg syndrome or prolactinoma [2,5,6]. These addictive behaviors include repetitive, stereotyped, and purposeless behaviors (punding, hoarding, wandering) or excessively performed goal-directed behaviors (gambling, shopping, eating or sexual activity). Importantly, the pathophysiology of these non-motor side effects as well as why they only affect a subset of subjects is still poorly understood, although clinical and epidemiological evidence points toward interactions between some pre-existing vulnerability traits, the type of antiparkinsonian medications, and the pattern end extent of neurodegeneration.

In this context, developing animal models and behavioral tasks that can recapitulate some of the features of addictive behaviors observed in patients is mandatory for the understanding of their pathophysiology and to provide a testbed for the preclinical evaluation of candidate drugs useful for their mitigation. Here, we used two behavioral tasks to evaluate the effect of a selective lesion of the SNc induced by viral-mediated human-mutated alpha-synuclein and of the treatment with the dopaminergic agonist PPX in triggering repetitive and compulsive behaviors that are relevant to those observed in patients.

Hoarding or food-carrying is a typical behavior displayed by most rodents including rats and mice. Thus, this hoarding task was chosen as it is a spontaneous behavior that involves repetitive sequences of actions that can be easily investigated in laboratory settings with minimal equipment and training. Moreover, this behavior is dependent on dopamine homeostasis, as shown with a disruption of the behavior following administration of haloperidol [21] or after 6-hydroxydopamine-induced lesion of the ventral tegmental area (VTA) and its reinstatement with administration of l-dopa in VTA-lesioned rats [20]. Here, we demonstrate that unlike for VTA neurons, degeneration of SNc neurons does not affect hoarding behavior in rats, suggesting that the dopaminergic control of this behavior essentially relies on VTA rather than on SNc neurons. We further show that chronic treatment with PPX significantly affects hoarding, as shown with an increased time spent in the food pellet zone and performing repetitive interactions with food pellets. Importantly, such changes occurred at the expense of hoarding itself, as evidenced with a marked decrease of the number of collected pellets. These results indicate that administration of PPX was able to deviate the goal of the behavior (food pellet hoarding in the home cage) towards repetitive and purposeless manipulations of food pellets, a behavior reminiscent of punding. In line with a lack of effect of SNc neurodegeneration on hoarding behavior, the effects of PPX were identical in sham and lesioned rats, indicating a main contribution of the drug for the expression of these repetitive behaviors. These results are in agreement with clinical observations of punding in patients treated with D3/D2 agonists for conditions that are not associated with dopaminergic neurodegeneration such as restless legs syndrome or prolactinoma [2,6] and highlights the usefulness of this simple test to assess the potential of dopaminergic drugs to trigger repetitive sequences of spontaneous behaviors.

The PTSA task allows for the evaluation of compulsive behaviors using a paradigm in which compulsive-like behavior is induced by a disruption of the association between the response feedback and the performance. As in a regular extinction procedure, when lever-press are not rewarded, rats increase excessive lever-presses followed by magazine entry (ELP-C). However, the signal attenuation elicits lever-presses that are not followed by magazine visit (ELP-U). This PTSA-induced excessive lever pressing behavior associated with uncompleted trials on test stage has been proposed to bear some similarity with compulsive behaviors observed in humans [14,22,25]. PTSA-induced excessive lever press is enhanced by the D1 antagonist SCH 23,390 and the D2 agonist quinpirole [22,23,26].

Whereas a combined dopaminergic lesion encompassing the SNc and ventral tegmental area (VTA) did not modify compulsive behavior in rats, PPX treatment in lesioned animals induced a strong emergence of compulsive behaviors [25]. In the present study, we restricted the lesion to the SNc in order to model early stages of PD dopamine denervation, when dopamine replacement therapy is initiated. As observed for combined VTA and SNc lesions [25], the SNc lesion alone or PPX treatment alone failed to increase PTSA-induced uncompleted trials, and only the combination of lesion and PPX treatment impacted this behavior.

Analysis of the number of ELP-U during the test phase as an index of compulsive lever-pressing indicated that degeneration of SNc neurons promoted the expression of compulsive-like behavior. Furthermore, analysis of the overall behavioral outputs during the test stage (ELP-C vs. ELP-U) revealed that the pattern of behaviors was different between groups. Indeed, only lesioned rats treated with PPX displayed a higher number of ELP-U than ELP-C, suggesting that the combination of nigrostriatal lesion and PPX treatment can affect the behavioral output towards the expression of compulsive-like lever-pressing (ELP-U) instead of responses classically observed after a regular extinction (ELP-C). However, unlike what was observed in rats with combined VTA and SNc lesion [25], PPX treatment following the SNc lesion did not enhance PTSA-induced ELP-U, suggesting that the pattern of dopamine denervation may play a critical role for the emergence of compulsive-like behaviors after treatment with dopamine agonists. In line with an involvement of the VTA in the expression of compulsive lever-pressing in the PTSA task, previous work demonstrated that chronic administration of quinpirole led to a decreased activity of VTA neurons, as shown with a reduced number of active neurons and decreased bursting activity that was correlated with behavioral outputs in this model [23]. Similar to quinpirole, chronic administration of PPX has also been shown to reduce spontaneous and bursting activity of VTA neurons in rats [27].

A single dose of PPX (0.3 mg/kg) was tested in this study based on its therapeutical relevance. It should be acknowledged that this single dose precludes any investigation of possible dose-dependent effects of PPX on the observed behavioral outputs. Also, an assessment of anxiety and impulsivity may have proven useful to understand how anxiety and decreased inhibitory control may represent vulnerability traits that could promote the emergence of perseverative and compulsive behaviors after treatment with dopamine agonists. In support of this hypothesis, outbred rats selected based on phenotypic traits (two-way active avoidance behavior) were found to display different behavioral responses to dopamine agonists [28,29,30].

Together with previous work using the same task [25], our results suggest that VTA degeneration combined to SNc dopamine loss may be required for PPX treatment to strongly induce the emergence of compulsive behaviors. This is in accordance with several imaging studies showing that PD patients with ICD exhibit reduced levels of dopamine transporter in the ventral striatum [31,32,33]. This is also corroborated by other studies that found a reduced dopamine synthesis capacity [34] and reduced activation of the ventral striatum at rest in patients with ICD [35]. According to a recently proposed vulnerability-stress model [36], such peculiar pattern of dopamine denervation may represent an intrinsic vulnerability that would alter the processing of action–outcome associations and reinforcement mechanisms when exposed to dopamine replacement therapy.

## Data Availability

The data that support the findings of this study are available from the corresponding author upon reasonable request.
